# Development of destructive thyroiditis and diabetes mellitus after three injections of pembrolizumab for skin melanoma

**DOI:** 10.14341/probl12698

**Published:** 2021-02-09

**Authors:** A. A. Glibka, G. A. Mel`nichenko, M. S. Mikhina, N. V. Mazurina, G. Yu. Kharkevich

**Affiliations:** Endocrinology Research Centre; Endocrinology Research Centre; Endocrinology Research Centre; Endocrinology Research Centre; N.N. Blokhin National Medical Research Center of Oncology

**Keywords:** immune checkpoint inhibitors, Pembrolizumab, thyrotoxicosis, hypothyroidism, diabetes mellitus, melanoma, clinical case

## Abstract

The exponential rise in the use of immune checkpoint inhibitors (Ipilimumab, Nivolumab, Pembrolizumab, Atezolizumab, Durvalumab, and Avelumab) as the new standard for cancer treatment increase the incidence the immune-related adverse events due to immune activation. Endocrine immune-related adverse events are the third most commonly reported. Thyroid gland is most susceptible to autoimmune dysfunctions from immune checkpoint inhibitors and associated with the use of anti-PD-1 monoclonal antibodies. Hypophysitis develops more often during therapy with anti-CTLA-4 monoclonal antibodies. But such immune-related adverse events as diabetes mellitus, hypoparathyroidism are rare (about 1% of cases).We present a clinical case of the patient with skin melanoma who was prescribed therapy with immune checkpoints inhibitors (Pembrolizumab). Immune-related adverse events developed with damage to the endocrine organs after 3 Pembrolizumab injections. Of greatest interest is the development of two endocrine immune-related adverse events at once: destructive thyroiditis (with a short phase of thyrotoxicosis and subsequent persistent hypothyroidism) and diabetes mellitus. We tried to reflect the chronology of diseases and their features as fully as possible for endocrinologists, oncologists, therapists, family doctors and other medical doctors of related specialties.

## Abstract (ru)

Экспоненциальный рост применения ингибиторов контрольных точек иммунного ответа (ипилимумаб, ниволумаб, пембролизумаб, атезолизумаб, дурвалумаб и авелумаб) в качестве нового стандарта лечения злокачественных опухолей приводит к увеличению случаев иммуноопосредованных нежелательных явлений (иоНЯ), обусловленных активацией иммунной системы. Эндокринные иоНЯ — третьи по частоте выявления. При этом щитовидная железа наиболее подвержена аутоиммунной агрессии, вызванной ингибиторами контрольных точек иммунного ответа, и ассоциирована с использованием анти-PD-1-моноклональных антител. В то время как гипофизит чаще развивается на фоне терапии анти-CTLA-4-моноклональными антителами. Такие иоНЯ, как сахарный диабет, гипопаратиреоз, поражения гонад, встречаются крайне редко (около 1% случаев).Мы представляем клинический случай пациентки с меланомой кожи, которой была назначена терапия ингибиторами контрольных точек иммунного ответа (препаратом пембролизумаб). На фоне данной терапии после 3 введений развились иоНЯ, в том числе с поражением эндокринных органов. Наибольший интерес представляет развитие сразу двух эндокринных иоНЯ: деструктивного тиреоидита (с короткой фазой тиреотоксикоза и последующим стойким гипотиреозом) и сахарного диабета. Мы постарались наиболее полно отразить хронологию заболеваний и их особенности для привлечения внимания эндокринологов, онкологов, терапевтов, семейных врачей и врачей других смежных специальностей.

## BACKGROUND

In 2018, James P. Allison and Tasuku Honjo were awarded the Nobel Peace Prize in Medicine for their discovery of a fundamentally new approach in the treatment of malignant neoplasms [1]. The antitumor effect of this type of immunotherapy is realized through blocking the control signaling pathways CTLA-4 (cytotoxic T-lymphocyte-associated protein 4), PD-1 (programmed cell death-1 protein) and PD-L1 (programmed cell death receptor ligand-1), which leads to an increase in the antitumor activity of the immune system and is accompanied by an improvement in overall survival rates in a number of tumors [2].

The study of the mechanisms of regulation of immune control led to the creation of a new class of immuno-oncological drugs — immune checkpoint inhibitors (ICPI). For the first time, ICPI was approved by the FDA in 2011 for the treatment of metastatic melanoma — an anti-CTLA-4 monoclonal antibody (ipilimumab). In 2014, the FDA approved two drugs from the subgroup of anti-PD-1 monoclonal antibodies (nivolumab and pembrolizumab), and already in 2016, three drugs from the subgroup of anti-PD-L1 monoclonal antibodies (atezolizumab, avelumab and durvalumab). In the Russian Federation, registration of all these drugs took place in 2016–2019 [3]. The range of oncological diseases for which the use of ICTIO is approved is continuously increasing.

The use of this type of antitumor immunotherapy is accompanied by the risk (through modulation of T-lymphocytes) of the development of immune-related adverse events (irAE). Endocrine irAEs in the frequency of detection occupy the 3rd place, yielding only to lesions of the skin and gastrointestinal tract [2, 4]. More rare types of irAEs are damage to the nervous system, liver, lungs and other organs. To date, there is an association between a certain subgroup of ICPI and the affected organ of the endocrine system. Thus, with the use of anti-CTLA-4 monoclonal antibodies, pituitary glands are more common (especially in elderly men), and with the treatment with anti-PD-1 monoclonal antibodies, thyroid dysfunction occurs. irAEs such as diabetes mellitus, hypoparathyroidism, and gonadal diseases develop extremely rarely, which does not allow at this stage to track their relationship with a certain subgroup of ICPI.

All irAEs are described in accordance with the CTCAE (Common Terminology Criteria for Adverse Events) classification from the mild degree (1) to extremely severe (4) and registration of a lethal outcome due to the pronounced irAEs (5) [5]. Endocrine irAEs of 1–2 degrees are more common [2].

Taking into account the severity of the manifestations of the main oncological disease, the erased clinical picture of irAEs [6, 7], a rapidly progressive course (in contrast to classical autoimmune diseases of the endocrine system) [8], irreversibility of the lesion [7, 8] and, in some cases of a life-threatening nature (for example, hypophysitis with the development of secondary adrenal insufficiency, myxedema coma, severe thyrotoxicosis, fulminant diabetes mellitus, adrenalitis) [6], timely detection of endocrine irAEs is necessary. A fully prescribed therapy makes it possible to continue the immunotherapy of ICPI of the underlying disease in full, and also improves the patient’s quality of life [6, 9].

The following is a clinical case of a patient who developed several irAEa after 3 intravenous injections of a drug from the subgroup of anti-PD-1 monoclonal antibodies (pembrolizumab) as adjuvant immunotherapy after removal of metastases of melanoma of the skin of the left foot in the inguinal lymph nodes (pT2aN1aM0 St IIIa, BRAF mut).

## CLINICAL CASE

Patient O., 34 years old, was admitted to the hospital of the National Medical Endocrinology Research Center with complaints to general weakness, unstable glycemic parameters (from 2.9 to 17.0 mmol/l), fatigue, thirst, episodes of hypoglycemia (3–4 times a week ) and flashes.

From the anamnesis (anamnesis morbi), it is known that in 2018, during the pedicure, a pigmented lesion was revealed in the heel area in the form of a black spot with bloody inclusions up to 0.3 cm in size. This lesion did not bother but gradually increased in size.

In April 2019, the patient noticed a tissue defect and bleeding (in the morning and while walking). Due to oncological alertness, I consulted a doctor. When examining the skin of the foot along the inner surface, it was determined: exophytic lesion which is multilayer, with the presence of hyperkeratosis, horny masses, actively bleeding when keratosis was removed; the edge was pigmented (hemosiderin in the mycelium), up to 6 mm in diameter. Cytological examination of scrapings from the surface of skin lesion showed blood, horny masses, few neutrophilic leukocytes and lymphocytes, cells of stratified squamous epithelium without atypia. In view of the data obtained (the absence of tumor cells in the papilla and a typical pigment network in the lesion, the presence of hyperkeratosis and layering of the process, the visual presence of hemosiderin in the mycelium), the process was considered as focal mycosis, and it was recommended to take antifungal drugs for 2 weeks.

However, in the following months, a slow increase in the exophytic component of the lesion was noted. In the summer of 2019, a total biopsy of the skin fragment of the left foot with ulceration and necrosis was performed, followed by histological confirmation of skin melanoma on the medial-plantar surface of the middle third of the left foot: epithelioid and non-cellular nodular melanoma of the foot skin with poor pigment, a large number of mitoses, expressed by lymphoid infiltration (the level of invasion according to Clarke 3–4, tumor thickness according to Breslow — 1.2 mm), tumor cells were not detected at the edges of resection. The second stage was re-excision of the postoperative scar with combined plasty of the defect with a skin flap on the vascular pedicle and a free skin flap from the upper third of the left thigh, as well as a sentinel lymph node biopsy. In 1 and 3 sentinel lymph nodes metastasis of a pigmented, epithelioid cell melanoma with high mitotic activity (12 mitoses per 1 mm2), 0.3 cm in the largest dimension, without extension out of the node capsule was revealed. The BRAF mutation c.1799T> A (p.V600E) was also detected in the tumor.

According to computed tomography (CT) of the thoracic cage, abdominal cavity and small pelvis, magnetic resonance imaging (MRI) of the brain, bone scintigraphy no data for distant metastases were obtained.

In autumn of 2019, the patient started adjuvant immunotherapy with immune checkpoint inhibitors. Three injections of pembrolizumab were administered once every 3 weeks. The patient tolerated the first injection of the drug (150 mg) satisfactorily but began to feel a lack of air (she constantly opened the windows for ventilation, went out into the fresh air, slept only with the windows open at night). After the 2nd injection (200 mg), she noted a deterioration in well-being after 16 days: severe headache, nausea, changes in appetite (hyporexia, then hyperorexia), a change in taste sensations (hypogeusia, then hypergeusia), weakness, drowsiness, turning into insomnia, increased fatigue, episodes of irritability followed by apathy. After the third injection of pembrolizumab (200 mg), the following symptoms were added to the intensified previously indicated symptoms: dry mouth and skin, polydipsia, visual impairment, itching in the vagina, difficulty concentrating. She independently measured glycemia — 15.3 mmol/L. Immunotherapy with pembrolizumab was canceled.

In December 2019, the patient was admitted to the endocrinological department of the regional hospital where she was diagnosed with type 1 diabetes mellitus, taking into account the presence of ketonuria +++, levels of glycated hemoglobin (HbA1c) — 7.5% and C-peptide — 0,01 ng/ml (1.1–4.4) and intensified basal-bolus insulin therapy (insulin glargine and aspart) was initiated. The patient was not trained, despite the titration of insulin doses, severe hyperglycemia persisted up to 18.0 mmol/L, and therefore the insulins were changed to degludec and glulisine at the place of residence. During hospitalization, thyrotoxicosis was also detected: thyroid stimulating hormone (TSH) 0.01 μIU/ml, free thyroxine (T4 free) 28.58 pmol/l, free triiodothyronine (T3 free) 6.54 pmol/l, antibodies (Ab) to thyroid peroxidase (TPO) 0.8 IU/ml (0–9.0), Ab to TSH receptor 0.45 IU/ml (0–1.75). Ultrasound of the thyroid gland: total volume 12.9 cm3, homogeneous structure, moderately reduced echogenicity, with color Doppler mapping (CDC) blood flow in the parenchyma is not changed, nodular lesions and enlarged lymph nodes are not revealed. The condition was regarded as thyrotoxicosis, and thiamazole was prescribed 10 mg in the morning and 5 mg in the evening.

At the same time, a follow-up examination was carried out for the main (oncological) disease. CT of thoracic and abdominal organs with intravenous contrast enhancement - no secondary pathology. Ultrasound: in the projection of the left heel in the area of the postoperative scar, an infiltrate (26 × 46 mm) with echo-negative inclusions was found, next to it was an identical infiltrate (up to 17 mm). Multispiral CT of the left foot: in the soft tissues of the left heel area, there is focus of induration is up to 28 × 16 × 17 mm. A biopsy of the lesion of the left calcaneal region was performed: in smears on the background of blood and structureless substance fragments of connective tissue, scattered few small monomorphic cells of a mesenchymal nature, probably of a fibroplastic series without signs of atypia — tumor elements were not detected within the delivered material. Ultrasound of soft tissues and peripheral lymph nodes: signs of slight hyperplasia of the left inguinal lymph node. Dynamic control was recommended.

Within 1 month the patient took the indicated dose of thiamazole which was then canceled: TSH 0.068 µIU/ml (0.27–4.2), T4 free 13.0 pmol/l (10.8–22), T3 free 3.56 pmol/l (3.1–6.8), AT to TPO 13.26 IU/ml (0–34).

Ultrasound of the thyroid gland: the total volume is 11.15 cm3; the structure is homogeneous, with single follicles (up to 15 mm), echogenicity is usual; no enlarged lymph nodes of the neck were found.

In February 2020, she turned to the NMIC of Endocrinology on an outpatient basis with complaints to significant fluctuations in glycemia during the day (from 2.9 to 17.0 mmol/L), and also provided the test results:

TSH 8.290 μIU/ml (0.27–4.2), T4 free 9.65 pmol/l (10.8–22), T3 free 3.43 pmol/l (3.1–6.8), AT to TPO 12 IU/ml (0–34) — the condition is considered as destructive thyroiditis, hypothyroid phase. The patient was hospitalized for follow-up examination, selection of the necessary therapy and training.

According to the anamnesis (anamnesis vitae), it is known that from early childhood there were difficulties with routine vaccination (the development of an allergic reaction in the form of a cough), then the asthmatic component gradually developed to dampness (observed by a pulmonologist). At 6 years old — adenoidectomy; in 2006 — an accident with a fracture of the left ankle (treatment — a plaster cast).

Allergic reaction to vitamins of group C (dermatitis); household chemicals (contact dermatitis); bloom, dust, dampness (rhinitis + asthmatic component).

Heredity: in the mother — endometrial hyperplasia during menopause, obesity grade 2; the father has chronic venous insufficiency (surgical treatment); grandmother (on the father’s side) — resection of the thyroid gland (for goiter), obesity grade 2, hypertension (HD), coronary heart disease (CHD), myocardial infarction (MI), type 2 diabetes mellitus (DM) (diet), pancreatitis (pancreatic necrosis is the cause of death); grandfather (paternal) — GB, ischemic heart disease, type 2 diabetes (gliclazide + diet), pancreatitis, MI (4 times, cause of death — extensive MI); grandmother (maternal) — GB, chronic heart failure, acute cerebrovascular accident; a cousin (paternal) — ovarian cancer (unilateral oophorectomy + chemotherapy), remission for more than 5 years; the great-uncle and grandmothers (on the father’s side) have type 2 diabetes.

Gynecological history — three births: 2003 — natural birth (healthy son, 60 cm, 4230 g); 2013 — natural childbirth (healthy son, 57 cm, 3960 g); 2016 — laparoscopic appendectomy at 22–23 weeks, then antenatal fetal death at 28–29 weeks, childbirth, stillborn son (histology results — leukocytic placentitis); there are no abortions.

On examination, weight 72 kg, height 162 cm, BMI 27.2 kg/m2. In the area of the postoperative wound (along the medial surface of the left foot arch), there is a pale pink scar without signs of inflammation, with signs of engraftment of the graft. The menstrual cycle is not broken, according to the words. Organs and systems — within norm.

In laboratory examination: НbА1с 8.3%; hemoglobin 104 g/l (112–153); hematocrit 32.6% (35–46); erythrocytes 3.76 × 1012 cells/l (3.8–5.2); C-peptide 0.01 ng/ml (1.1–4.4); TSH 2.493 mIU/l (1.1–4.4); creatinine 65.4 μmol/l (50–98) — glomerular filtration rate (GFR) according to the CKD– EPI formula 105.73 ml/min/1.73 m2; Ca total. 2.3 mmol/l (2.15–2.55); total cholesterol 4.7 mmol/l (3.3–5.2); low density lipoproteins (LDL) 3.079 mmol/l (1.1–3); high density lipoproteins 1.143 mmol/l (1.15–2.6); triglycerides 0.72 mmol/l (0.1–1.7).

Until hospitalization at the National Medical Endocrinology Research Center, autoantibodies to pancreatic antigens were not investigated, so it was decided to conduct a serological blood test for differential diagnosis of the type of diabetes (Table 1) [10].

**Table:** Table 1. Antibodies to pancreatic β-cells

Parameter	Results, U/ml	Referenc range, U/ml
Ab to IAA	2.59	0–10
Ab to GAD	700	0–10
Ab to IA-2	<1	0–10
Ab to ICA	15	0–1
Ab to ZnT-8	<10	0–15

Notes: Ab to IAA — antibodies to insulin; Ab to GAD — antibodies to glutamate decarboxylase; Ab to IA-2 — antibodies to tyrosine phosphatase; Ab to ICA — antibodies to surface antigens; Ab to ZnT-8 — antibodies to the zinc transporter. Thus, the autoimmune genesis of diabetes mellitus was confirmed.

The patient was also consulted by a cardiologist. According to the HM-ECG data, 18,000 single monomorphic ventricular extrasystoles (ST-T without diagnostically significant dynamics) were registered, the administration of metoprolol succinate 25 mg in the morning was recommended.

During hospitalization, the selection of the dose of prolonged insulin, as well as carbohydrate coefficients and insulin sensitivity factor, was carried out, individual training was carried out on the basic principles of achieving target glycemic indicators. Thanks to careful monitoring of glycemia (9 times a day), the phenomenon of “morning dawn” was revealed, and insulin glulisine at a dose of 1 U was additionally prescribed (Table 2).

**Table:** Table 2. Glycemic profile during hospitalization, mmol/l

Day	Before breakfast	2 hours after breakfast	Before dinner	2 hours after dinner	Before supper	2 hours after supper	21:00	03:00	06:00
1	–	9.3	4.9	7.2	6.3	4.6	4.7	6.2	10
2	11.3	7.5	3.6	9.3	10.9	–	12.0	11.9	17.4
3	14.4	11.1	9.4	4.8	3.4	10.7	9.5	5.6	9.6
4	9.9	5.6	4.6	8.4	8.3	11.9	11.7	6.3	6.4
5	7.3	–	–	–	–	–	–	–	–

Considering the development of diabetes mellitus [6, 8, 9, 13–15] as an irAE after ICPI therapy (in our case, pembrolizumab), it was decided to encode diabetes according to ICD-10: E13.9. Other specified forms of diabetes mellitus without complications.

The final diagnosis was formulated as follows: Diabetes mellitus due to therapy with immune response inhibitors for skin melanoma. Destructive thyroiditis, hypothyroid phase, drug compensation. Melanoma of the skin of the left foot pT2aN1aM0 St IIIa. Excision from 17.07.2019, 21.08.2019 — re-excision of the postoperative wound on the skin of the left foot with combined plasty of the defect (a flap on a vascular pedicle and a free skin flap) with a sentinel lymph node biopsy (SLNU). Condition against the background of immunotherapy with pembrolizumab from 18.10.2019 — 3 sessions. Mutation BRAF V600E. Mild anemia. Individual target level HbA1c <6.5%.

At discharge from the hospital, the patient was recommended: insulin degludec 23–25 U at 8.00; insulin glulisine 4–12 U (carbohydrate coefficient: 1 XE: 2 U before breakfast, 1 XE: 1.5 U before lunch, 1 XE: 1 U before dinner, 1 XE: 1.5 U after 21.00; additionally 1 U in 5.00; IPF (insulin sensitivity factor) 2.5 mmol/L/U); levothyroxine sodium 50 µg (TSH control after 4–6 weeks); metoprolol succinate 25 mg in the morning; self-monitoring of glycemia at least 4 times a day; quantitative assessment of carbohydrates in the system

«Bread units»; describes the regularity of dynamic examination, as well as additional examination for identified anemia and dyslipidemia.

She was repeatedly consulted by oncologists about the need to resume adjuvant therapy with pembrolizumab — given the lack of progression of the underlying cancer, it was decided to refrain from using ICTIO.

Within 1 month, she took metoprolol succinate 25 mg in the morning, then after another 1 month, the XM-ECG was repeated and recorded: 506 single monomorphic ventricular extrasystoles, 7 episodes of bigeminy (ST-T without diagnostically significant dynamics) — therapy was canceled.

4 months after the hospitalization we described and 7 months after the last infusion of pembrolizumab, patient O. developed vitilig-like hypopigmentation under the lower lip and in the perineal region.

At the time of writing the clinical case, patient O. achieved near-target glycemic levels: HbA1c 7.5% (Fig. 1).

**Figure:**
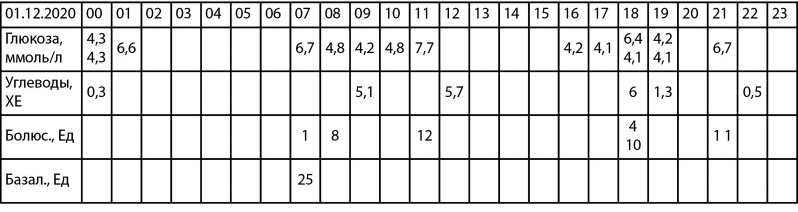
Figure 1. Glycemic profile per day

Hypothyroidism is medically compensated: TSH 2.1 μIU/ml (0.27–4.2), T4 free 16.45 pmol/l (10.8–22), T3 free 4.22 pmol/l (3.1–6.8). The dose adjustment of levothyroxine sodium was performed on an outpatient basis (Table 3 and Fig. 2). For the treatment of iron deficiency anemia, therapy with an oral iron-containing drug is carried out: hemoglobin 120 g/l (120–180), hematocrit 34.1% (36–56), erythrocytes 3.94 × 1012 cells/l (3.8–5.3); serum iron 12.8 µmol/l (7–31).

**Figure:**
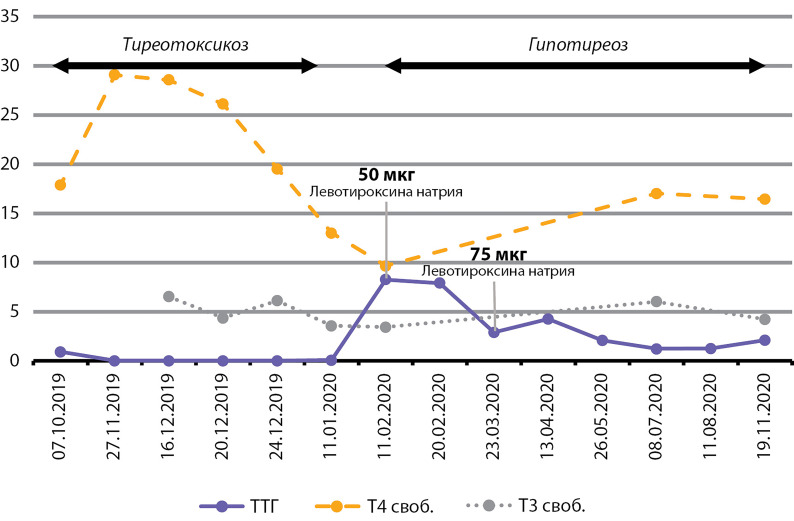
Figure 2. The course of destructive thyroiditis

**Table:** Table 3. Results of hormonal studies of thyroid function before the first administration of pembrolizumab and until the description of the clinical case

Date	TSH, µIU/l	Free Т4, pmol/l	Free T3, pmol/l	Drug treatment
07.10.2019	0.91	17.88	-	
27.11.2019	0.016	29.09	-	
16.12.2019	0.01	28.58	6.54	Thiamazole 10 mg in the morning and 5
20.12.2019	0.01	26.13	4.36	
24.12.2019	0.005	19.52	6.13	
11.01.2020	0.068	13	3.56	Thiamazole withdrawal
11.02.2020	8.29	9.65	3.43	Levothyroxine sodium 50 µg
20.02.2020	7.93	-	-	
23.03.2020	2.9	-	-	Levothyroxine sodium 75 µg
13.04.2020	4.26	-	-	
26.05.2020	2.08	-	-	
08.07.2020	1.225	17.02	6.03	
11.08.2020	1.26	-	-	
19.11.2020	2.1	16.45	4.22	

## DISCUSSION

In the clinical case described by us, a hyperergic immune response can be observed, which led to the development of several irAEs with simultaneous manifestation. However, if in the case of vitiligo in melanoma we can talk about the formation of a good response to ICPI (a possible increase in the sensitivity of melanocytes [2]), and hence an increase in overall and relapse-free survival [11], the reason for the development of endocrine lesions is unclear. However, such vitilig-like depigmentation differs from classical vitiligo (the latter is localized in photo-exposed areas, and the Kebner phenomenon is observed) [12].

The median onset of endocrine irAEs is determined in the range of 7–20 weeks [6] from the first administration of ICPI. In our patient, the first pronounced hyperglycemia was detected at the 6th week from the start of immunotherapy: before each injection of the drug, a biochemical blood test was performed and normoglycemia (5.0 mmol/l) was observed. Thyrotoxicosis was diagnosed simultaneously with diabetes mellitus. At the same time, before the first administration of pembrolizumab, the function of the thyroid gland was not impaired (TSH 0.91 μIU/ml, free T4 17.88 pmol/l).

It should be noted that the first line in the treatment of a wide variety of irAE is the appointment of glucocorticoids. Supraphysiological doses required to control the side process can potentially cause or aggravate existing hyperglycemia. Therefore, it is justified to control blood glucose with each ICPI administration and treatment for any other irAE [6, 13]. The initiation of insulin therapy in patients with immune-mediated diabetes is not in doubt, the management is carried out according to accepted standards and clinical guidelines [6].

The decision to prescribe thyrostatics to such patients, despite laboratory-confirmed thyrotoxicosis, is legitimate upon confirmation of Graves’ disease [6]. The most common variant of thyroid pathology when using ICPI is destructive thyroiditis with transient thyrotoxicosis [6, 8, 14], and it would be advisable to prescribe glucocorticoid therapy.

The appointment of thyrostatics in such patients (with laboratory diagnosed thyrotoxicosis) is not recommended [6] before imaging diagnostic methods (ultrasound of the thyroid gland, scintigraphy) and confirmation of the development of Graves’ disease: it is possible that the cytolytic process and thyrotoxicosis worsen.

Before prescribing levothyroxine sodium in patients who have undergone or are undergoing ICPI therapy, in the presence of nonspecific symptoms (severe weakness, drowsiness, vomiting, etc.) or indirect biochemical laboratory data (hyponatremia, hypoglycemia), it is desirable to determine blood cortisol in the morning [6, 8, 9] to exclude primary (extremely rare) or secondary (more frequent with the development of hypophysitis) adrenal insufficiency. In the described case, no signs of hypocorticism were observed.

A similar clinical case was presented by Hakami in 2019 [13] — the development of type 1 diabetes and hypothyroidism in a 52-year-old man during monotherapy with pembrolizumab. At the same time, the first damage to the thyroid gland (thyrotoxicosis, TSH 0.09 mIU/l) developed after the second injection of the drug, followed by the formation of persistent hypothyroidism (anti-TPO antibodies negative) after the fifth injection of pembrolizumab. Diabetes mellitus with severe diabetic ketoacidosis developed 2 weeks after the seventh infusion of anti-PD-1 monoclonal antibody with a characteristic clinical picture (nausea, vomiting, polyuria, polydipsia) and biochemical parameters (glucose 38.6 mmol/l, ketones 4.9 mmol/l (<0.6), C-peptide <0.01 μg/l (1.1–4.4), HbA1c 8.3%), but negative serological — Ab to GAD and AT to ICA were given). Upon reaching the stabilization of the state (hormonal therapy), due to the presence of metastases and a good response to the drug, pembrolizumab therapy was continued.

The use of 2 ICPI, aimed at different targets of exposure (ipilimumab + anti-PD-1- or anti-PD-L1 monoclonal antibody), induces a synergistic antitumor response, which means that IONN can occur more often [7, 13, 14]. Thus, in another 52-year-old patient with metastatic melanoma [11], a combined immunotherapy regimen (ipilimumab + nivolumab) was used. Three injections of these drugs were performed, after which ICPI had to be canceled, since the patient developed hypophysitis with the formation of hypopituitarism (deficiency of TSH, FSH, LH, PRL, as well as cortisol and testosterone), insipidus and diabetes mellitus. At the same time, antibodies to β-cells of the pancreas (Ab to GAD, Ab to IA-2, Ab to ZnT-8 were tested) were negative, C-peptide 0.05 nmol/l (0.4–1.5), НbА1с 7.7%.

Thus, taking into account the variability and specificity of clinical manifestations of irAEs, each patient should be informed about the signs of the most formidable endocrine irAE (hypophysitis, diabetes mellitus, hypoparathyroidism), and also draw the attention of patients to the need to inform the attending physician about all new symptoms.

In contrast to thyroid lesions, which can develop with a frequency of up to 50%, the incidence of diabetes mellitus as an irAE is extremely low [8]. Nevertheless, it should be remembered that it is possible to develop irAE with damage to several endocrine glands at the same time. Such clinical observations should be analyzed in detail both in order to identify possible patterns and to create practical recommendations for specialists faced with this problem in their clinical practice.

## CONCLUSION

The possible development of irAE when using ICPI therapy dictates the need for a more attentive attitude towards such patients. Due to the severity of the condition, the clinical picture can be erased, which means that it can lead to untimely diagnosis of endocrinopathies.

Endocrine irAEs more often correspond to 1–2 degrees according to CTCAE, however, timely initiated therapy not only prevents the development of complications, but also makes it possible to continue immunotherapy of the underlying cancer in full, which increases overall and relapse-free survival.

It is important for practice to search for genetic, serological, or biochemical markers that will make it possible to predict the development of endocrine ions, as well as to better understand the mechanisms of ICPI effect.

## References

[cit1] NobelPrize.org. [Internet]. The Nobel Prize in Physiology or Medicine 2018. [cited 2020 Nov 30]. Available from: https://www.nobelprize.org/prizes/medicine/2018/summary/

[cit2] Shubnikova E. V., Bukatina T. M., Velts N. Yu., Kaperko D. A., Kutekhova G. V. (2020). Immune Response Checkpoint Inhibitors: New Risks of a New Class of Antitumor Agents. Safety and Risk of Pharmacotherapy.

[cit3] Grls.rosminzdrav.ru. [Internet]. [cited 2020 Nov 30]. Available from: https://grls.rosminzdrav.ru/grls.aspx.

[cit4] Mazarico I., Capel I., Giménez-Palop O., Albert L., Berges I., Luchtenberg F., García Y., Fernández-Morales L. A., De Pedro V. J., Caixàs A., Rigla M. (2019). Low frequency of positive antithyroid antibodies is observed in patients with thyroid dysfunction related to immune check point inhibitors. Journal of Endocrinological Investigation.

[cit5] CTCAE v5.0 incorporates certain elements of the MedDRA terminology. For further details on MedDRA refer to the MedDRA MSSO. [Internet]. [cited 2020 Nov 30]. Available from: https://www.meddra.org/.

[cit6] Higham C E, Olsson-Brown A, Carroll P, Cooksley T, Larkin J, Lorigan P, Morganstein D, Trainer P J, _ _ (2018). SOCIETY FOR ENDOCRINOLOGY ENDOCRINE EMERGENCY GUIDANCE: Acute management of the endocrine complications of checkpoint inhibitor therapy. Endocrine Connections.

[cit7] Ferrari Silvia Martina, Fallahi Poupak, Galetta Fabio, Citi Emanuele, Benvenga Salvatore, Antonelli Alessandro (2018). Thyroid disorders induced by checkpoint inhibitors. Reviews in Endocrine and Metabolic Disorders.

[cit8] Yudin D. I., Laktionov K. K., Sarantseva K. A., Borisova O. I., Breder V. V., Reutova E. V., Beloyartseva M. F., Kruteleva S. Yu., Dzhanyan I. A. (2020). Immuno-related endocrinopathy in patients treated with immune checkpoint inhibitors. Meditsinskiy sovet = Medical Council.

[cit9] Pigarova Ekaterina A., Dzeranova Larisa K., Nuralieva Nurana F., Mel`nichenko Galina A. (2018). Diagnosis and treatment of endocrinological complications of immunotherapy of oncological diseases. Obesity and metabolism.

[cit10] Dedov Ivan I., Shestakova Marina V., Mayorov Aleksandr Y., Vikulova Olga K., Galstyan Gagik R., Kuraeva Tamara L., Peterkova Valentina A., Smirnova Olga M., Starostina Elena G., Surkova Elena V., Sukhareva Olga Y., Tokmakova Alla Y., Shamkhalova Minara S., Jarek-Martynova Ivona Renata, Artemova Ekaterina V., Beshlieva Diana D., Bondarenko Olga N., Volevodz Natalya N., Grigoryan Olga R., Gomova Irina S., Dzhemilova Zera N., Esayan Roza M., Ibragimova Liudmila I., Kalashnikov Viktor Y., Kononenko Irina V., Laptev Dmitry N., Lipatov Dmitry V., Motovilin Oleg G., Nikonova Tatiana V., Rozhivanov Roman V., Shestakova Ekaterina A. (2019). Standards of specialized diabetes care. Edited by Dedov I.I., Shestakova M.V., Mayorov A.Yu. 9th edition. Diabetes mellitus.

[cit11] Nardin Charlée, Jeand'heur Anne, Bouiller Kévin, Valnet-Rabier Marie Blanche, Dresco Flora, Castagna Julie, Mareschal Adrien, Carlet Clémentine, Nerich Virginie, Limat Samuel, Puzenat Eve, Aubin François (2019). Vitiligo under anti–programmed cell death-1 therapy is associated with increased survival in melanoma patients. Journal of the American Academy of Dermatology.

[cit12] Gracia-Cazaña T, Padgett E, Hernández-García A, et al. Vitiligo-like lesions located over In-transit metastases of malignant melanoma as a clinical marker of complete response to pembrolizumab. Dermatol Online J. 2019;25(12):12. 32045170

[cit13] Hakami Osamah A, Ioana Julia, Ahmad Shahzad, Tun Tommy Kyaw, Sreenan Seamus, McDermott John H (2019). A case of pembrolizumab-induced severe DKA and hypothyroidism in a patient with metastatic melanoma. Endocrinology, Diabetes & Metabolism Case Reports.

[cit14] Barroso-Sousa Romualdo, Barry William T., Garrido-Castro Ana C., Hodi F. Stephen, Min Le, Krop Ian E., Tolaney Sara M. (2017). Incidence of Endocrine Dysfunction Following the Use of Different Immune Checkpoint Inhibitor Regimens. JAMA Oncology.

[cit15] Gunawan Florence, George Elizabeth, Roberts Adam (2018). Combination immune checkpoint inhibitor therapy nivolumab and ipilimumab associated with multiple endocrinopathies. Endocrinology, Diabetes & Metabolism Case Reports.

